# Clinicians’ Experience of Collaboration in the Treatment of Suicidal Clients Within the Collaborative Assessment and Management of Suicidality Framework

**DOI:** 10.1177/00302228211020579

**Published:** 2021-05-30

**Authors:** Bríd Fogarty, Sharon Houghton, Eoin Galavan, Páraic S. O’Súilleabháin

**Affiliations:** 1 Department of Psychology, University of Limerick, Ireland; 2 Health Service Executive, Dublin, Ireland; 3 Health Research Institute, University of Limerick, Ireland

**Keywords:** suicidality, suicidal intervention, interpretative phenomenological analysis, clinicians, Collaborative Assessment and Management of Suicide

## Abstract

**Introduction:**

There is little known about the clinicians’ experience of collaboration using the Collaborative Assessment and Management of Suicide (CAMS) framework. This study aimed to give voice to the clinician experience.

**Method:**

A qualitative design utilised semi-structured interviews with ten psychologists who worked in a Suicide Assessment and Treatment Service (SATS) in Ireland which utilises the CAMS framework.

**Results:**

An Interpretative Phenomenological Analysis (IPA) approach revealed several important findings. The superordinate themes included ‘Finding Safety’, ‘Regulation of the Self’, ‘Connecting’, and ‘Systemic Challenges’.

**Discussion:**

The CAMS framework plays an important role in providing a safe base for the clinician (in terms of understanding suicidality, in addition to the structures of the framework). It provides a mechanism in which to process difficult emotions, and a way of communicating a formulation of suicide to the treating team. Importantly, the CAMS emerged as facilitating a collaborative, therapeutic way of working.

Globally, at least 800,000 people die by suicide each year and approximately twenty times more attempt suicide annually (World Health Organization [WHO], 2017). Suicide was the reported cause of 1.4 per cent of all deaths in 2016, making it the eighteenth leading cause of death worldwide (WHO, 2017). In Ireland, provisional figures from 2018 (Hse, 2019) indicate that there were 352 suicides reported in Ireland in 2018. This aligns with a modest decline in reported suicides in recent years, with 383 deaths by suicide reported in 2017 and 506 deaths in 2016 (Hse, 2019). It is estimated that suicidal attempts occur at a ratio of 1:20 for every completed suicide (WHO, 2017). Approximately 45 per cent of people who complete suicide consult their GP one month prior to death without disclosing suicidal ideation or intent (Isometsa et al., 1995). In Mental Health care, it is reported that almost one quarter of people who have died by suicide engaged with mental health services in the 12 months before their death (Appleby et al., 1999). In their examination of 153 Irish post-mortem reports of people who died by probable suicide between January 2006 and May 2012, Kielty et al. (2015) found that approximately 40 per cent had had prior contact with mental health services. Perhaps more interestingly, analysis of the associated toxicology reports noted that less than one-third of those who had been prescribed with psychopharmacological medication by mental health services were not taking their medication at the time of death. This suggests a high rate of non-compliance with medication intervention (Kielty et al., 2015). While those identified as at risk of dying by suicide are often admitted to residential psychiatric hospital facilities, it is estimated that approximately five per cent of all suicides occur by people admitted to psychiatric inpatient services (Walsh et al., 2015). Jobes (2016) highlights that there is little research evidence that hospitalisation is an effective response to suicidality. It is clear that interaction experiences with services are crucial in terms of the care and support offered to clients presenting with suicidality in order to support them to cope.

## Psychotherapeutic Interventions for Suicidal Behaviours

In Ireland, clients presenting with suicidal thoughts or behaviour typically find themselves referred to Mental Health teams. However, it is increasingly evident that interventions which centre on the psychiatric diagnosis rather than suicidality as the fundamental difficulty have been found persistently ineffective in reducing suicidal behaviours (Beasley et al., 2007; Cuijpers et al., 2013). Very few psychotherapeutic inventions have demonstrated a repeated RCT evidence-base (Brown & Jager-Hyman, 2014). Such evidence-based interventions include dialectical behaviour therapy (DBT), cognitive therapy for suicide prevention (CT-SP), brief cognitive behaviour therapy (B-CBT), and the Collaborative Assessment and Management of Suicidality (CAMS) (Jobes, Piehl, et al., 2018). In 2000, a number of clinical suicidologists convened in Aeschi, Switzerland, to address their concerns regarding contemporary clinical approaches to the suicidal client (Jobes, 2016). Since then, the Aeschi group has influenced a number of emerging evidence-based approaches (Gysin-Maillart et al., 2016; Jobes, 2016; Jobes, Piehl, et al., 2018). Thus, there are a number of common elements to psychotherapeutic interventions, including the phenomenological approach to understanding the client’s experience of suicidality, and specialised skill development to meet the needs of the suicidal client. In addition, there is a shared focus on the therapeutic alliance, collaborative approach, and the psychotherapeutic principles of validation, empathy, and relatedness (Jobes, 2016; Jobes & Ballard, 2011; Schechter & Goldblatt, 2011).

## Collaborative Assessment and Management of Suicidality

The Collaborative Assessment and Management of Suicidality (CAMS) was developed by David Jobes (2016) as a suicide-specific therapeutic framework for working with suicidal clients. To this end, it adopts the stance of “suicidality as the central clinical problem, independent of diagnosis” (Jobes & Drozd, 2004, p. 74), and exclusively focuses on the idiosyncratic experience of suicidality as a coping mechanism whilst unapologetically aiming to enhance the client’s reasons for living. As part of the philosophy of care is to provide the intervention within in a community setting rather than hospitalisation, there is an initial emphasis on safety and self-harm risks (Jobes, 2006). Procedurally, the clinician, sitting beside, and in collaboration with the client, is guided by the Suicide Status Form (SSF) for assessment, generation of treatment plan, ongoing tracking of suicidal risk, and ensuring focus is maintained on difficulties related to the client’s experience of suicidality (Jobes, 2016). The SSF contains Likert rating scales and open-ended questions which explore the client’s experience of psychological pain, stress, agitation, hopelessness, self-hatred, and overall risk of suicide (Jobes, 2016). Jobes, Gregorian, et al. (2018) stress that the CAMS is conceptualised as a “philosophy of care” (p. 244) that orientates the clinician towards understanding the underlying “drivers” of client suicidality. In this way, any subsequent therapeutic intervention is open to the clinical techniques, therapeutic orientations, intervention strategies, and theoretical approaches which the clinician introduces in response to individual client needs (Jobes, 2016). Each subsequent CAMS session begins with a completion of the SSF and sessions continue until the suicidality resolves. Resolution is operationally defined as three consecutive sessions of no reported suicidality.

The CAMS approach symbolises a radical shift in terms of the deliberate and ongoing collaborative approach with clients in the context of suicidality (Galavan & Repper, 2017). Supporting the therapeutic alliance is the clinician’s position that suicidality is an understandable (although maladaptive) resolution which serves as a functional coping mechanism for the client. On such a basis, Jobes asserts that the clinician is better placed to explore alternative, adaptive coping strategies which meet the client needs (Jobes, 2015). Fundamental to the CAMS approach, is a phenomenological aspect that encourages the clinician to understand the client’s suicidality (Jobes, 2016). There is a strong focus on the development of the therapeutic alliance and utilising this relationship as the intervention mechanism (Jobes, 2011). Jobes maintains that the therapeutic alliance is supported by purposefully engaging the client as an active participant in the assessment process and in supporting them to co-author treatment plans (Jobes, 2011). Galavan and Repper (2017) noted that the philosophy of this framework attempts to avoid the latent coercive interactions that can infect clinician-suicidal client interactions wherein the suicidal client is conceptualised as “threat and trouble”, to be controlled or avoided in order to circumvent blame or legal consequences. Jobes maintains that the SSF supports the clinician-client dyad in their shared understanding and co-authored intervention plan for the identified suicidal drivers (Jobes, Gregorian, et al., 2018).

As previously mentioned, CAMS is one of few suicidality interventions which has a strong empirical evidence base, including correlational studies (Arkov et al., 2008; Jobes et al., 1997, 2009; Nielsen et al., 2011), studies of open trial (Ellis et al., 2012), non-randomised control case-control (Jobes et al., 2005), controlled comparisons (Ellis et al., 2015; 2017) and randomized control trials (Andreasson et al., 2016; Comtois et al., 2011; Jobes et al., 2017).

While evidence mounts for the efficacy of the CAMS, much less is known about the clinician’s experience of this novel approach. (Jobes, Piehl, et al., 2018) proposes the CAMS as a potential remedy to the clinical challenges that arise in the therapeutic assessment and intervention of suicidality. However, there is a paucity in qualitative research with regard to the CAMS and as such, little is understood about the clinician’s experience of the CAMS or if it differs from other literature regarding psychotherapeutic work with suicidal clients.

## Therapeutic Alliance With Suicidal Clients

The therapeutic alliance is widely accepted as the cornerstone to effective work with clients who are suicidal (Bostik & Everall, 2007; Jobes, 2011; Leenaars, 2006; Michel, 2011; Michel et al., 2002; Schechter et al., 2013). Therapeutic empathy with the suicidal wish or “the death wish” is considered necessary in order to form a genuine connection and quality therapeutic alliance with the suicidal client (Orbach, 2001, p. 166). There is a general consensus that therapeutic work with clients expressing suicidal intent can have an adverse impact on the clinician and how they work with clients expressing suicidality (Pearlman & Saakvitne, 1995; Reeves, 2010; Reeves & Mintz, 2001). Weinberg et al. (2011) proposed fifteen alliance-interfering factors and five alliance-facilitating factors. Some of the alliance interfering and potentially destructive factors include the wish to die, projection of suicidal intent, pervasive shame, painful life experience, disturbed attachment and chronic hopelessness. Suicidal clients often present in therapy with experiences of intense shame about their suicidal thoughts and behaviours, which inhibits their ability to talk about these with the clinician (Jobes, Piehl, et al., 2018). As a result, intense transference and countertransference responses can be triggered. Transference and countertransference responses of distrust, self-blame and hopelessness can be triggered between the clinician and the suicidal client (Jobes, Piehl, et al., 2018; Leenaars, 1994).

In an influential paper in the field of suicidology, Maltsberger and Buie (1974) proposed a countertransference theory unique to suicidal clients. They suggested that the clinician who is not accepting of suicide will likely experience aversion, malice and anger towards the client because of their suicidality and their ambivalence towards intervention (Milch, 1990). Firestone (2014) argues that passive anger encompassed the most common emotion provoked in clinicians whilst working with clients expressing suicidality. These feelings obstruct the clinician’s ability to empathise with the client, which ultimately undermines the therapeutic process (Orbach, 2001; Richards, 2000). This gives rise to potential for rupture to the alliance.

The client’s ability to die by suicide invariably introduces struggles of control, power and vulnerability into the therapeutic dyad (Jobes & Ballard, 2011). The clinician can experience immense pressure to prevent the client from completing suicide, which runs the risk of creating an adversarial rather than collaborative relationship (Jobes & Ballard, 2011). The fear of making mistakes or being blamed can result in defensive clinical practice, such as admitting a person to inpatient care, becoming coercive or controlling in the therapeutic relationship, limiting the exploration of the client’s phenomenology of suicidality or taking limited clinical risks that may benefit the client (Jobes, 2016). In this way, the possibility for working collaboratively is diminished significantly as the client’s stance in the dyad is pushed out of focus in order to manage the clinician’s anxieties.

The clinician’s sense of responsibility was also prominent in the literature as a factor that can impact on therapeutic alliance. Jobes et al. (2000) argue that aspects of blame and interpersonal responsibility are imbedded in every suicide. The degree to which the clinician feels responsible for the client impacts on the therapeutic alliance, the efficacy of the intervention, and personal impact on the clinician (Whitfield, 2011).

## Research Question

In order to work effectively with suicidality, it is clear that such challenges must be met and overcome. To this end, the broad research question developed as a means of remaining open to all aspects of the CAMS clinician experience is as follows:

*“What are CAMS clinicians’ experiences of collaboration?”*

## Materials and Methods

### Interpretative Phenomenological Analysis

Interpretative Phenomenological Analysis (IPA) is a qualitative research approach in psychology that is committed to exploring how people generate meaning about a phenomenon they have experienced (Smith et al., 2009), through a process of detailed reflective analysis (Peat et al., 2019). IPA is informed by concepts and considerations from philosophy that are rooted in three principals: phenomenology, hermeneutics, and idiography. This provides a useful framework for researchers’ understandings of how individuals make sense of their personal and social worlds (Smith et al., 2009).

### Recruitment

This study utilised a purposive homogenous approach for recruiting participants. Criteria for eligibility were that participants must be psychologists who had completed the CAMS training and had applied this approach to working with clients expressing suicidality. Participants were recruited from the Health Service Executive (HSE) East area of the Republic of Ireland. All participants currently or had previously worked in a Suicide Assessment and Treatment Service (SATS) where the CAMS approach is utilised. The study information sheet was circulated by e-mail to a pool of psychologists who had completed the CAMS training and who currently or had previously worked in the specialised suicide service using the CAMS approach. In total, 10 psychologists expressed interest in participating in the study. All met the inclusion criteria for the research and were invited to participate in an interview. Participants were recruited to the study between August 2018 and December 2018.

### Participants

All the participants in this study were clinical psychologists who had at least five months of experience of working in the suicidal specific service. Participants were eight females and two males. During the interviews, participants indicated they had been working with clients who presented as suicidal (with various populations) for time spans varying from two to 12 years (M = 6; SD = 2.89). Participants also estimated the amount of clients they had worked with who had been suicidal. This ranged from five to 40 clients per participant (M = 25.78; SD = 15.05).

### Procedure

Prospective participants who received the information sheet by email were encouraged to contact the lead researcher by email to organise a suitable time and date to conduct the interview. During this email exchange, participants were provided with a copy of the consent form to read through prior to the interview. Interviews followed a broad structure of developing rapport with the participant, answering any questions they had about the study, clarifying how confidentiality and anonymity would be maintained, participants were reminded that they were not under any obligation to participate and were free to withdraw from the study at any point. A semi-structured interview was conducted using an interview schedule designed by the researcher prior to data collection. The process of developing the schedule was a dynamic, iterative one that involved exploration of previous literature and reflection on the broader research question. The questions were developed in line with the principles outlined in Smith et al. (2009). Interviews were recorded and interview times ranged from 35 minutes 31 seconds to 77 minutes 52 seconds (M = 59.53 minutes; SD = 11.95). On conclusion of the interview, participants were offered the opportunity to review their transcript in the following weeks.

Reflective field notes were kept by the researcher after each interview and throughout the data collection process. These focused on subjective thoughts and emotive responses to content and interview experiences, such as first impressions, reflections and initial interpretations of the participant’s account. These were referred to during analysis and supported the analysis process.

Each interview recording was transcribed by the researcher into a Microsoft Word document. The IPA analytic process is defined by a dynamic, iterative and inductive cycle that moves between the particular and the shared in addition to the individual and the whole (Smith et al., 2009). Analysis occurred over an extended period of time. Significant efforts were made to become familiar with the data; interview recordings were listened to multiple times in order to become familiar with the participant’s idiom and ‘voice’. In addition, interview transcripts were read and re-read, and the process was reflected upon. The analytic steps outlined in Smith et al. (2009) were followed. Consistent with Smith et al. (2009), Lucy Yardley’s (2000) four broad principles for validity in qualitative research were applied in this study. These are ‘sensitivity to context’, ‘commitment and rigour’, ‘transparency and coherence’ and ‘impact and importance’ (Yardley, 2000).

### Ethical Considerations

Ethical approval for this study was obtained from the local Hospital Research Ethics Board. Appropriate measures were taken during the design of this study to mediate for the following ethical considerations: making initial contact with participants, vulnerability of participants as staff/employees, data storage and management and potential distress during interview.

## Results

Four superordinate themes and nine subordinate themes emerged from the participant’s narratives. These are outlined in Table 1.

**Table 1.** Summary of Superordinate and Subordinate Themes.

Superordinate theme	Subordinate theme
Finding safety	“*I’m doing my best”* (Following the CAMS)
	Feeling supported
Regulation of the self	*“Checking into yourself”* (Attuning to the Self)
	*“It’s worrying. It’s scary”* (Difficult Emotions)
Connecting	“Into the Person’s World”
	Establishing a Shared Way of Working
Systemic challenges	Tension and Battles with the medical model
	Finding My Voice
	Challenges of the CAMS

## Finding Safety

This theme embraces the participants’ description of seeking and finding their own bases of safety and the associated freedoms this affords in terms of working with clients who are suicidal.

### “I’m Doing My Best” (Following the CAMS)

Grounding personal anxiety in the structure and processes of the CAMS framework was a feature of almost all the participant narratives. Participants revealed that they found the organized process of the CAMS containing for anticipatory anxiety before sessions with clients. Containment stemmed from reassurance that as clinicians, they had drawn on a framework grounded in up-to-date evidence base.

 … you feel like you’re doing the best you can […] like I was doing a reasonable job based on this and am … and like that helps you with the anxiety because you’re kind of thinking like there’s nothing else I can do that I know about or that is reasonable within this. (Bronagh, p. 81)

The perception of the CAMS as “*very thorough and everything exists within the frame of the CAMS” (- Bronagh p.5)* allays worries of being ‘enough’ and tolerating the inherent uncertainty encompassed in the work. Narrative elucidates a sense of active competency in the work and focusing on the establishing a possible way forward for the client. In completing the assessment, participants spoke about the experience of being confident to ask questions which evoked fear, being comprehensive in the assessment of suicidality, and finding safety in the thoroughness of the process.

you are asking the difficult questions—but being able to do so in a way that’s, am, bringing the person on board. It just it feels … I don’t know. It feels safer generally. (Ciara, p. 10)

The introduction to the CAMS model appears to have been a transformative learning experience for participants, as Aiden highlights his experience of psychological models of suicidality being largely unknown in clinical practice:

Not in any of the four degrees that I have done. Not in any of the many supervisors that I’ve had. Not in any article that I’d ever looked up for myself had I ever come across this. It sounds really ridiculous to me at this point … and it was a very vivid learning moment that I remember … you know they say, “the penny drops”? This was more like the bank vault dropped!”[laughs] I was in a training session about CAMS and about half an hour into it I went “Oh, my god! Oh yes! Why did I never think of this? Why have I never looked this up?” I never knew to. I never knew to look up psychological models of suicide. (Aiden, p. 27)

### Feeling Supported

Feeling supported by peers and colleagues was a compelling theme for nearly all participants’ in terms of feeling grounded, contained, and in enhancing resilience for working with persons expressing suicidality. Participants placed value on the opportunity to share vulnerabilities encountered in their work with peers and to have that responded to reciprocally. It is likely this provides an experience of validation and competence. Participants spoke about “*having somewhere*” (Aiden, p. 29) that gives a sense of security, responsiveness, and acceptance of vulnerabilities. In addition, participant’s discussed how the support they feel from their teams impacts on the felt sense of safety and confidence.

that kind of reduces that sense of anxiety. It means that it is shared, ah, shared sense of responsibility and I can more easily park it at the end of the day. (John, p. 12)

This speaks to the pervasiveness of the sense of responsibility and also the immense value of team support for participants.

## Regulation of the Self

This superordinate theme encompasses values held by all the participants within their narrative of being curious and attuned to their personal and emotional inner narratives when working with clients who are suicidal.

### “Checking Into Yourself” (Attuning to the Self)

This subtheme captures all the participant’s experiences of thinking about and reflecting on their own internal processes.

Participant’s attributed engagement in reflective practice as a mechanism for enhancing the ability to engage collaboratively in the CAMS process with clients.

If you’re triggered [ … ] whatever it is that you’re experiencing emotionally to the extent that that’s your own stuff, and, am, if you’re not aware of that, that interferes with empathy then. That interferes with collaborative working because you know you’re not responding to the person with empathy anymore, or you are kind of responding to your own emotional state which is heightened. (Deirdre, p. 30)

Maintaining an awareness of the emotional demands of overall caseloads and available resources before entering the therapy was important for participants.

 … you have to … be aware of what you’re bringing into the room and your own emotional state at that point and, am, sometimes that can be a challenge, depending […] on your workload and making sure that you’re not overly burnt out. (Hannah, p. 30)

*“Overly burnt out”* is an interesting turn of phrase since it infers that there is a correct or acceptable level of burnout. It perhaps speaks to a familiarity with the experience, either personally or through a colleague.

Participants demonstrated the struggle with the philosophical right to self-determination while simultaneously adhering to the professional concept of *duty of care*. Questions such as “*how much I will go,* ‘*oh no, actually we really need to hospitalize you now*.’” (Grainne, p. 16) gives us a sense of ambiguity in the philosophical thoughts about suicide.

Of particular interest is participant description of the biological feedback of adrenaline and feelings of heroism that come from engaging with risk based work. Some participants acknowledged and accepted their presence but were thoughtful about managing these sensations.

It’s awful work. It’s really hard but there is a little bit of a ‘we’re on the war zone—we’re soldiers’ and there’s almost like a badge you get, you know? … Again you have to be mindful of all of these emotions that are popping up when you’re working with people - that they’re not running the show. But they’re definitely there. (Isabella, p. 40)

The image of the war zone is affecting, illustrating the life and death nature of the work, but also the heightened fear system that drives that image.

### “It’s Worrying. It’s Scary” (Difficult Emotions)

While there is a strong super-ordinate theme of findings safety in the participant’s narratives, difficult emotions remain part of the narratives throughout and merit commentary. Anxiety and overwhelming worry is expressed throughout, where the awareness of potential heavy outcomes that can engage the clinician. Participants spoke to the instinctive challenge of the cognitive and emotional dichotomy of accepting another person’s suicidal wish, whilst theoretically understanding it.

In theory I can accept that it might not work and they might, am, they still might take their own life, but the reality is a very hard thing to actually accept. (Grainne, p. 6)

Participant’s spoke of an intense experience of responsibility for the life of another person. Images striking fear, isolation, burden, and disconnectedness from the wider clinical team permeates the narratives supporting this theme.

It’s worrying. It’s scary. Psychologically it’s hard to deal with. Am, it feels like a lot of pressure and responsibility on, am, our shoulders. Am, and when you’re one to one in a room with somebody it feels like it’s on your shoulders, even though other members of the team may feel it’s on their shoulders. (Aiden, p. 16)

A striking image of the cliff-edge is provided providing emphasises a sense of constant threat where one’s every move is significant. He speaks about his awareness of potential outcomes and the pressure to engage his client in the, presumably, life-saving intervention. This analogy gives rise to a progressive sense of responsibility to intervene and the responsibility to be successful in that endeavour.

There is a sense at which the, am, the immediacy of the risk can enter into the room, you know, am. The sense that you’re working on a cliff-edge, you know. (John, p. 14)

Participants identified the intensity to their susceptibility to personal pain and distress, illustrating how the self is deeply embedded in the therapeutic process and thus the potential for harm to the self is ever present.

It’s a really vulnerable … it’s a really vulnerable place to be in. It’s a really vulnerable to…it’s really vulnerable to work with people who are suicidal. (Fiona, p. 37)

Anxiety about being “enough” is a challenge for participants. This questioning of being enough speaks to a deep-seated vulnerability and questioning of worth. Participants discussed feeling de-skilled when the anticipated progressive decline in suicidal ideation fails to occur in the work, and the subsequent inward projection of blame. This subtheme underscores the intensity of the felt sense of personal and professional responsibility in the participant narratives, with the worst case scenario consciously held by all participants.

## Connecting

This superordinate theme refers to the participant’s experience of connectedness and collaborative working with their client when using the CAMS framework.

### “Into the Person’s World”

Each of the participant’s spoke of the experience of connecting authentically with their clients and empathising with their suicidality. It is clear from the narratives that the theoretical models synthesised by the CAMS framework facilitates this connection process.

 … one of the things that this allows is for us to walk away from being frightened of suicide and frightened of death and into … into the people’s world. This is where the person is at. (Aiden, p. 63)

Participants’ outlined how the process facilitated individualised meaning making for the client in completing the assessment. Placing value on the time spent doing the individualised suicide-specific assessment with the client was identified as facilitative to feeling attuned to the client’s suicidal experience

 … you get down to the, am, to the drivers, and you can validate and empathize with those and really understand how much pain someone is in, and then it kind of becomes easier. (Grainne, p. 29)

Participants convey an emotional acceptance of the client’s current suicidality and recognition that this is the starting point of their therapeutic relationship. One participant’s experience of the CAMS differed from the other participants, describing the forms and paperwork implicated in the CAMS as a barrier to the therapeutic relationship.

### Establishing a Shared Way of Working

This theme refers to establishing the understanding of engaging in a collaborative manner of working together. For psychologists this is often a process of socialisation and modelling for the client. For some participants this process identified a number of key challenges whereby there are positioned by the client in a role of expert in the relationship which indicated a subordinate role for the client. This serves to reinforce the participants sense of responsibility.

But, I suppose her working model was a bit different to mine, […] this person was saying, ‘that is not my experience; I want you to be the expert; and I need you to tell me what to do’. (Aiden, p. 13)

As well as the dynamic challenges, participants’ also spoke of experiences that appear to be validating for them as clinicians in terms of their clients feeling heard, understood, and appreciative of the shift in their experience of working with professionals.

How does this work? Okay, oh right, you’re actually trying to understand me. (Eimear, p. 26)

Descriptions of how participants engaged clients in the process is indicative of holding the client’s resilience in mind by reminding them of their personal agency. They stressed their expertise in their life experiences and their control.

A big thing about the treatment plans is not, like, what you think they want, […]it’s, you know, ‘What is the thing that’s going to make the difference in your life?’ (Fiona, p. 41)

Participants described feeling the team approach is *“very much, it feels very much equal. That the two of you are in it together” (Hannah, p. 5).*

For one participant, the experience is quite different. She reflected that her experience of utilizing the CAMS in establishing a collaborative working alliance is not *“hugely, hugely, different” (Deirdre, p. 9)* to her engagement in other therapeutic approaches.

## Systemic Challenges

The fourth superordinate theme which emerged from the participant’s narratives describes overcoming the challenges experienced in the systems encircling the therapeutic work.

### Tension and Battles With the Medical Model

All of the participants alluded to the tension of the power dynamics within services wherein Psychiatry or the medical model of care holds a position of authority. A common thread throughout participant accounts was the various experiences of compromise with medical leadership of the team.

how the team views suicide and whose job it is to manage risk, am, is a big issue (Fiona, p. 74).

Participants attributed the decision-making tensions which arise to risk adversity and to a degree of uncontained anxiety held by Psychiatry owing to their position of perceived accountability:

 … and I would say that the psychiatrists in the teams would still regard themselves as holding the clinical risk. (Deirdre, p. 55)

Participants revealed the position of compromise they hold as psychologists in a hierarchical, medical-model team and the impact on their own practice, such as in the use of CAMS. They also draw attention to the considerable effort and tensions of *“lots of battles to fight”(Grainne, p.19)* that they experience, not only in relation to the conceptualisation and intervention of suicidality.

Participants outlined frustration with the team’s hypervigilance and fear-driven team reactions to the person’s safety without exploring the client’s potential for resilience or supporting them to be active in their management of suicidality.

“ … a culture of maybe an over-reliance on admitting people who have a suicidal thought and maybe not too much of a team decision on how we should approach the person. (Hannah, p. 16)

### Finding My Voice

In the context of team tensions regarding the dominating medical model and the projected identity of Psychologists being CAMS practitioners, participants have found ways of nurturing an understanding of suicidality in their teams.

Taking a proactive approach, participants spoke about subtly conveying key pieces of information that they would like the team to take on broad in terms for understanding suicidality as distinct from pathological discourse.

We can almost, possibly out of self-protection, de-personify the person, um, into categories such as ‘depression; and, um, ‘risk of death; and ‘suicidal ideation’ but … as … let’s keep this person’s life and their story and their view and their wish and their skill and their experience and everything that they have going on; let’s keep that as part of our conversation because that is really what is going to work. (Aiden, p. 64)

Participants illustrated mentoring roles they have taken with other interested MDT members who are tentatively utilising the CAMS framework in their practice. Taking an opportunity to model the theoretically sound understanding of suicidality, some participants ensured that the Psychologists in Clinical Training on placement with the team utilise the CAMS framework in their client work and discuss this at team meetings. Participants found that the MDT responded positively to watching the Psychologists in Clinical Training acclimatise quickly to the framework and demonstrate positive experiences through discussion at team meetings.

I find the CAMS so useful, and particularly useful in terms of, am, clearly being able to report to the team around, am, you know, assessment and intervention around suicidality as well. (John, p. 29)

### Challenges of the CAMS

The participants’ experiences of the challenges of the CAMS were varied but the strength of viewpoints was noted and merited commentary. Throughout her narrative Deirdre remained adamant that the CAMS framework was not a good fit with her therapeutic approach. There were a number of aspects that Deirdre found lacking, in particular the relationship and collaborative way or working.

I think that the relationship is lacking in it, that’s what I would feel … to me, the paperwork always felt like a bit of a barrier as well, you know. (Deirdre, p. 26)

She believed it merely created a mechanism for holding the psychologist’s own anxiety but did not serve to enhance the experience for the client. Her opinion is in stark contrast to the collaborative and containing experiences articulated by the other participants and there is a sense that for Deirdre the CAMS relationship feels superficial and solely preoccupied with suicidality. She perhaps feels she is dismissing the holistic needs of the client by discharging them to the waitlist; a tension she clearly expressed regarding the centrality of the therapist-client relationship to her practice. Nor is Deirdre alone in her concerns about the paperwork; most participants revealed that completing the forms presented challenges for a small number of clients.

 … on the occasions where a person will refuse maybe to complete CAMS maybe because again because of literacy issues or, am, education issues, or others issues that might just drive to not want to use a form. (Aiden, p. 61)

## Conclusions

All participants in the study spoke about finding the CAMS as a source of safety in their work. This theoretical understanding provided clinicians with a frame of reference to conceptualise suicide and also a mechanism in which to process their own experiences. This is in line with Brown et al. (2011) who have suggested that theoretical models of suicidality have a therapeutic role in creating a shared understanding. It’s reasonable to suggest that in this instance, the theory is providing a containing, therapeutic frame for the clinician’s personal process.

In relation to the structure and organisation of the framework that participant’s valued, it is noted in DBT literature, where Miller et al. (2011) found lower levels of cortisol and self-reported stress in DBT clinicians in comparison to controls. The authors concluded that this may be in part due to the structural elements of therapy, such as clear intervention goals and team consultations. Whilst most participants felt contained by the structure and process, concerns were also raised in this study that the CAMS may be functioning to meet the emotional and intellectual needs of the clinician and not specifically the clients.

Participant narratives in this study focused on describing the benefit of having colleagues and team members that are interested in and/or use a psychological understanding of suicide. For participants, this described a meaning greater than solely validation and support. It extended to a learning culture and skill development. In DBT research, Swales (2010) describes a training functioning to the structured team consultation process of DBT intervention. Swales noted that development of skills in this team environment maintains clinician’s resilience in working with the challenges and complexities of clients presenting with BPD.

A narrative shared amongst participants was being attuned to their own internal processes in terms of what they were bringing to the work (Attuning to the Self) and what was being triggered by the work (Difficult Emotions). It is clear that reflection plays a critical role in their work. This is illustrated in the literature where reflective practice is gaining more attention and there is encouraging support for benefits of this practice (Mann et al., 2009). Of interest, Schön (2017) described that reflective practice often functions as a framework from processing difficult or complex issues that clinicians face, but their training did provide them with the skills to deal with it.

Participant narratives discussed being mindful to avoid burnout, by remaining attentive to work demands and the personal resources required when working with persons who are suicidal. This is mirrored in the research, Leenaars (1994) suggests that a clinician’s caseload must be limited in terms of suicidal clients, noting the impossibility of providing effective care without risking burnout or emotional detachment from the demands of the work.

Participants in this study spoke about the intensity of adverse emotions that can be triggers by the suicidal client. These emotions included anxiety, responsibility, fear, pressures, isolation, worry, vulnerability, feeling self-critical, unskilled and not feeling “enough”. These findings are supported by the academic literature in relation to working therapeutically with suicidal clients. Anxiety and worry are referenced widely in the literature (Gurrister & Kane, 1978; Porter, 2013; Reeves & Mintz, 2001), as is professional responsibility and pressures (Anderson, 2000; Moody, 2010; Porter, 2013; Ting et al., 2006), fear and vulnerability (Jobes, Piehl, et al., 2018; Reeves & Mintz, 2001; Roose, 2001) and self-questioning (Moody, 2010). What’s notable about the findings of this study is that negative emotions are directly internally for the clinician. Whilst other studies have reported aversion, malice and anger (Maltsberger & Buie, 1974; Milch, 1990; Reeves & Mintz, 2001), not one participant spoke about negative emotions towards their client. From the participant narratives, this is likely the results of a number of factors. Firstly, participant narratives illustrated a strong conceptual framework of suicidality as a function of unbearable distress which facilitated empathy. Secondly, the participants spoke about finding safety in the CAMS process which facilitated the ability to accept the client where they are at and be curious to their experiences. The findings from this study illustrate that intense difficult emotions remain in the therapeutic work but appeared to be more muted than reported in other literature, and negative countertransference was not reported in this study.

This understanding of the function of the suicidal wish appeared to facilitate genuine empathy for the client and connectedness in the relationship. This is in line with the fundamental approach promoted by the Aeschi working group (Michel, 2011) and the CAMS (Jobes, 2016); which views the suicidal thoughts, sensations and behaviours as actions that contain personal meaning for the client.

Of interest, participants in this study spoke about taking time to socialise clients to the collaborative, shared way of working. This is illustrated in the literature in the original academic writings of Bordin (1979) in terms of agreeing treatment goals, agreeing tasks and developing the personal bond. Indeed, Horvath and Symonds (1991) proposes that a strong alliance is frequently the outcome of negotiation.

Participants’ narratives in relation to team tensions were all in some form of compromise within a medical model system. Within this theme, participants’ narratives conveyed collective frustration and activation of their threat/fear system in some interactions within the dominant medicalised model of care. Indeed Orbach (2001) cautioned that the phenomenological approach to suicidality is a challenge for clinicians and teams trained to view suicidality with a defensive lens. For participant’s their experiences of didactic dynamics, at team and organisational levels, triggered emotive responses of frustration, feeling unheard, invalidated and dismissed. Of interest, this dynamic is similar in description to the literature of coercive, defensive and risk adverse practices when the clinician, or in this case the team, feels pervasive fears regarding blame and responsibility.

It must be noted that one participant felt very strongly that the CAMS was deficient with regard to the relationship element. This participant felt there was a superficial connection with the client’s distress as the distress was only addressed to the point of reduced risk. Indeed, this is discordant with the Aeschi approach where acknowledging that suicide has a past and is not determined solely by the present (Michel, 2011).

## Strengths and Limitations

This study aimed to address the evident paucity in the research exploring clinician experiences of working with clients who are suicidal. The findings offer valuable insights and expands on previous research (Gaffney et al., 2009; Moore & Donohue, 2016; Reeves & Mintz, 2001). In addition, it contributes generally to the field of clinical suicidology. While this study did comprise of several strengths which will inform future research, several limitations must be noted. A potential limitation of the present study was the variance in experience of the participant sample, ranging from two to 12 years (M = 6). As such, a broader range in order to differentiate experience within this context would be important. It must be acknowledged that the findings of this study are not necessarily representative of the views of other psychologists utilising the CAMS and the small sample size means that findings cannot be generalised. Using Interpretative Phenomenological Analysis generated in-depth and rich narratives around the experience of using the CAMS with clients who are suicidal. It facilitated analysis of meanings embedded in the data and signposted original opportunities for further research. There is a clear dearth of qualitative research in the literature and growing concerns that research in suicide has become subsequently stagnant (Hjelmeland & Knizek, 2010). This current study responds to this appeal for qualitative research to provide insight and understanding that may be helpful for suicide intervention.

## Implications for Clinical Practice

Firstly, the importance of finding safety was a strong theme in this analysis, finding safety through peer and team support. This involved having a structured, psychologically safe space for reflection in the team, akin to peer supervision. This is not part of the CAMS intervention, but the availability of this reflective peer support was particularly useful for clinicians in regulating their emotions and also feeling supported in their individual work. Whether this kind of support can develop into a structured element of the CAMS framework is uncertain, but it is clear that this is a helpful mechanism when working with such a challenging client group. Alternatively, the findings of this study have shown that this is happening naturally in some teams and it is perhaps a support that can be introduced and prioritised by individual teams. Secondly, establishing a shared understating of suicide was found to be a strong theme for all participants, resulting in individual efforts to model, mentor and informally disseminate information about theoretical understandings of suicide and the CAMS framework for intervention. Given the radically different understandings of suicide from medical model and psychological approach, there is a clear need for multidisciplinary mental health teams to develop a shared understanding of suicide and their roles and values in relation to suicide. Training and education in relation to suicide generally was a striking finding of this study, as the CAMS for many participants served as their first introduction to psychological models of suicide. Introducing suicide-specific education in professional training programmes for clinicians could be one way of promoting this understanding. Indeed, such reflections need to be prioritised at an organisational level. The findings of this study clearly show that when working with clients who are suicidal, clinicians are hypervigilant to the pressures of the wider system. Fear regarding blame, professional reviews and being thought of as incompetent by peers are very real pressures for clinicians and teams. This appears to be poorly dealt with by the wider mental health service currently. It is clear from the findings of this study that clinicians require a supportive, safe base in order to engage in effective, collaborative therapeutic work.

## Future Research

As the CAMS is considered appropriate for any discipline, it would be interesting to explore the experiences of other professionals’ (nursing, occupational therapy, social work, psychiatry) of using the CAMS and working therapeutically with clients who are suicidal. A number of challenges in using the CAMS were found in this study, for example overcoming client rejection of the paper and pencil SSF work. This may be interesting to explore in further detail from both the client’s and clinician’s perspectives. A feature that emerged in this study’s findings was the report of adrenaline driven biofeedback that was ignited when working with risk and is associated with feelings of honour and credit. This could be worth exploring with clinicians in terms of identity and motivation in their work with people who are suicidal.
